# Oswaldo Gonçalves Cruz: archives, memory and history

**DOI:** 10.1590/0037-8682-0313b-2020

**Published:** 2020-08-21

**Authors:** Dalmo Correia, Ana Luce Girão Soares de Lima, Cláudio Tadeu Daniel-Ribeiro

**Affiliations:** 1 Disciplina de Doenças Infecciosas e Parasitárias, Departamento de clínica Médica, Universidade Federal do Triângulo Mineiro, Uberaba, MG, Brasil.; 2 Departamento de Arquivo e Documentação, Casa de Oswaldo Cruz, Centro de Documentação em História da Saúde, Rio de Janeiro, RJ, Brazil.; 3 Laboratório de Pesquisa em Malária, Instituto Oswaldo Cruz, Pavilhão Leônidas Deane, Rio de Janeiro, RJ, Brasil.

Article presentations are usually made in editorials signed by editors who justify the publication, under the journal’s Editorial policy describing the perceptions generated by the manuscript since the awareness of the text until the definitive decision to publicize it. If written by the authors, the presentation may describe the rationale for writing the manuscript and the motivations for submitting it to that journal. It is not trivial that such a presentation is made jointly by editor and authors. Invited by Correia, D. Editor of the *Revista da Sociedade de Medicina Tropical (RSBMT) / Journal of the Brazilian Society of Tropical Medicine*, who thought they would do a better job - because they knew the history of the theme’s choice and the rationale for the text elaboration - Daniel-Ribeiro, C.T. and Lima, A.L.G.S, authors of “*Oswaldo Gonçalves Cruz: the character the scientist, the academician*”, imposed on the Editor the condition that he joined them in the task. Although difficult to manage and face, such a challenge would allow a broader scope and completeness in describing the reasons for that particular article being in that precise journal, and what to expect from the text. *RSBMT* strictly follows the rules and protocols - for organizing and structuring the papers it publishes - stablished by its Editorial Board and the Scientific Electronic Library Online (SciELO) for indexing scientific journals. No longer having the possibility to publish opinion articles or historical reviews and having restrictions on the object, structure and extension for the insertion of a text as an editorial or a major article in a regular number, it became unusual for *RSBMT* to consider articles on great figures of medicine, public health or biomedical research for publication. About two months ago, however, *RSBMT* received an informative and easily readable article describing picturesque or unknown aspects on the personal biography, scientific trajectory and academic life (at the Brazilian *Academia Nacional de Medicina-ANM*) of Oswaldo Cruz, perhaps one of the most prominent and well-known figures in the history of tropical medicine in Brazil and worldwide.

Taken to the writing of the text for the reasons that will become clear in the lines below, the authors opted to have it published in English, but in a Brazilian journal, since, although interested in reaching a repertoire of readers as vast and comprehensive as possible, they wished to do so in a journal that would be easily accessible to the Brazilian community of parasitologists, tropicalists and infectiologists. *RSBMT* was honored by the choice, but it felt confronted with the challenging task of publishing an article that, although welcome, comprised 16 pages of a dense text with 20 bibliographic references, 17 notes and 18 photographs. It demanded from the RSBMT Editor creativity and time for obtaining an agreement with *Scielo* to finally come to light in the form of a supplement preceded by this presentation.

The origin of the authors' motivation for writing the article traces back to the Scientific Session of *ANM*, held on August 3, 2017^1^. The session’s theme “Oswaldo Cruz: Researcher and Sanitarist” payed homage to the Academician in the centenary of his death (February 11, 1917) on the eve of his birthday date (August 5, 1872), when we celebrate the National Health Day in Brazil. To talk about the two aspects of Oswaldo Gonçalves Cruz's life, the *ANM* Board invited two scientists from the *Fundação Oswaldo Cruz (Fiocruz)*:Dr. Paulo Gadelha and Dr. Cláudio Tadeu Daniel-Ribeiro, former President of *Fiocruz* and former Director of the *Instituto Oswaldo Cruz (IOC),* respectively. Determined to publish the text referring to his speech, and having benefited from conversations held with and material provided by the researcher Ana Luce Girão Soares de Lima (*Casa de Oswaldo Cruz, COC-Fiocruz*), a specialist on the character’s biography, for the presentation at *ANM*, CTDR invited her to expand the text as co-author of the work.

We must also comment on the importance of the sources that allowed us to offer the readers a great deal of precise information and many historical pictures, some of them only made available because of the existence of the historical Archives of *ANM* and *COC-Fiocruz*.

The Oswaldo Cruz's biography is closely associated with the history of *Fiocruz* since its creation and the various transformations that this institution underwent in the early 20th century. Since 1900, when it received the name of *Instituto Soroterápico Federal,* in the midst of a serious health crisis, its main mission was the production of sera and vaccines. In 1908, when it was renamed the *Instituto Oswaldo Cruz*, its vocation for research and teaching, manifested since the first times of the *IOC* existence, became clearly consolidated, while the production activities increased and the provision of health services as well as the dissemination of science became main Institutional missions progressively. The Oswaldo Cruz Archive^2^ allows us to follow both paths: that of the institutional history and that of its patron’s trajectory, Oswaldo Cruz.

The archival history of this fund presents a couple of gaps, on which we do not have information, but some milestones help us to trace it back, even though with some time jumps.

There is, therefore, a large set of institutional documents, with special reference to the first letter from May 25, 1900, which provides us with the date of the beginning of the work in Manguinhos-RJ. It is reasonable to assume that this institutional documentation was accumulated by Oswaldo Cruz during the period in which he was at the head of the institution, from its beginning until 1916, when, due to illness, he was forced to leave the office. His trajectory at the head of the General Directorate of Public Health, between 1903 and 1909, is also very well represented, as well as his urban projects, when he was appointed mayor of the city of Petrópolis-RJ, in the mountainous region of Rio de Janeiro, after leaving *IOC*.

This set of information remained at the *Instituto Oswaldo Cruz* after the death of O. Cruz, and was organized by the archivist Albino Antonio Taveira during the 1940s. In the early 1970s, as part of the celebrations of the centenary of Oswaldo Cruz’s birthday, the museologist Luiz Fernando Fernandes Ribeiro, under the guidance of the head of the Library of Manguinhos, Lucília Meyer Friedmann, drew up a list of the documents, which contained material of personal nature, such as letters, notes and minutes. In this way, they could be opened again for public consultation. In 1990, the documents were placed under the custody of the Department of Archives and Documentation of the *Casa de Oswaldo Cruz*. However, its date range (1889-1972) encompasses a period earlier than the creation of the *Instituto Soroterápico* and extends beyond the date of Oswaldo Cruz's death. This is due to several shipments of documents made by family members, among which are those that were under the custody of Roberto Marinho de Azevedo Neto and Stella Oswaldo Cruz Penido, descendants of the scientist. In its current conformation, the Archive has 2.5 meters of documents including correspondences, reports, catalogs, notebooks, newspaper clippings, among other types, constituting an important source for research on the history of Oswaldo Gonçalves Cruz. It is worth mentioning in this set an important and significant series of correspondences that gathers letters, telegrams, cards and tickets shared by O. Cruz with his wife and children, as well as with Brazilian and foreign scientists, adding up to about two thousand items that cover almost three decades (1889-1916).

For these reasons, the Oswaldo Cruz’s Personal Archive of *Fiocruz* was registered in 2007 in the National Registry of Brazil of the UNESCO Memory of the World Program, whose main objectives are to ensure the preservation of documentary collections of world importance, disseminating these collections and democratizing access to documents.

Another source for obtention of historical material on Oswaldo Cruz was the Sergio D'Avila Aguinága Archive of the *ANM* ^3^. According to the *ANM* itself^4^, it was created in 1829 to “contribute to the study, discussion and development of the practices of medicine, surgery, public health and related sciences, in addition to serving as a consultative body of the Brazilian Government on health and medical education issues”. In its 191 years of existence, *ANM* has consolidated an information-rich (~ 1km of document long) archive available for consultation not only by doctors, health professionals and historians, but also by the general public interested in the Brazilian medicine and science history and even in major political and social events.

The Archive, officially founded in 2005 and named after its greatest organizer and supporter - the former Academic President Sergio D'Avila Aguinága, became an independent unit, representing the administrative structure for preservation of the institutional and the Brazilian medicine memories. The collection preserves textual, iconographic, audiovisual and sound documents recording the activities of the institution, as well as the functions and actions performed by its members and by other science and medicine personalities^3^.

The article “*Oswaldo Gonçalves Cruz: the character the scientist, the academician*”, by Daniel-Ribeiro & de Lima represents a significant work of historical search and rescue of the medical, scientific and academic trajectory of Oswaldo Gonçalves Cruz, one of the most important characters and precursors of the Brazilian public health and experimental medicine. It seems mandatory that, when presenting and justifying its publication in this issue of the RSBMT Vol.:53:(Supplement I), we acclaim the role that Institutions such as the Brazilian *Academia Nacional de Medicina* and the *Fundacão Oswaldo Cruz* play in organizing and maintaining scientific and medical memory archives that allow access to information and the construction of records that enrich the cultural and historical heritage of Brazil and humanity.
